# Left ventricular M‐mode prediction intervals in 7651 dogs: Population‐wide and selected breed‐specific values

**DOI:** 10.1111/jvim.15914

**Published:** 2020-10-02

**Authors:** Lilith Carla Esser, Martin Borkovec, Alexander Bauer, Jens Häggström, Gerhard Wess

**Affiliations:** ^1^ Clinic of Small Animal Medicine LMU University Munich Germany; ^2^ Statistical Consulting Unit StaBLab LMU University Munich Germany; ^3^ Department of Clinical Sciences, Faculty of Veterinary Medicine and Animal Science Swedish University of Agricultural Sciences Uppsala Sweden

**Keywords:** body weight, canine, echocardiography, heart dimensions, prediction intervals

## Abstract

**Background:**

Echocardiography is a common method to measure heart size in dogs. The heart dimensions are influenced by body weight (BW) and potentially by breed.

**Objectives:**

To establish BW‐dependent prediction intervals (PIs) of the left ventricular (LV) linear dimensions in a population of dogs of many breeds in multicenter environment, and to identify breeds deviating from these intervals.

**Dogs:**

Seven thousand six hundred and fifty‐one dogs.

**Methods:**

Retrospectively, data from heart screens conducted between 2009 and 2016 were included. Cardiac dimensional PIs were generated using allometric scaling including all nonsighthound dogs and values were compared to previously published PIs. The values measured in dogs of respective breeds, including sighthounds, were then compared to the overall nonsighthound PIs to identify deviant breeds. The interobserver‐variability of the measurements was determined using the explained residual variance.

**Results:**

Prediction intervals for the nonsighthound dogs were in agreement with previously published cardiac PIs, although the upper limits of the generated PIs of our study were slightly below those currently applied (except the interventricular septum in systole and the left ventricular free wall in diastole below 10.0 kg and 15.0 kg, respectively). Values measured in the nonsighthound breed Newfoundland deviated for most dimensions. Most of the sighthound breeds analyzed had greater cardiac dimensions, with the exception of the Irish Wolfhound.

**Conclusion and Importance:**

Findings of our study reinforces the value of BW‐dependent PIs for cardiac dimensions in dogs and suggest that these PIs are valid for most nonsighthound breeds, but not the sighthound breeds.

**Abbreviations**
BW: body weight
CC: Collegium Cardiologicum e.V.
FS: fractional shortening
GAM: generalized additive model
IVSd: interventricular septum in diastole
IVSs: interventricular septum in systole
LV: left ventricle
LVDd: left ventricular diameter in diastole
LVDs: left ventricular diameter in systole
LVWd: left ventricular free wall in diastole
LVWs: left ventricular free wall in systole
MM: M‐mode
PI: prediction interval
RVa: residual variance

## 1 INTRODUCTION

Cardiac disease is commonly diagnosed in dogs presented to primary care veterinary practices.^1^ Echocardiography is a commonly used method to measure the dimensions of the cardiac chambers and walls. Thus, it provides important information for establishing diagnosis and assessing disease severity.^2^ Cardiac dimensions are frequently measured using M‐mode (MM) and two‐dimensional (2D) echocardiography, where the left ventricular (LV) dimensions are measured using either method.^3^ The left ventricle (LV) dimensions are either measured in the right parasternal long or short axis views.^3^ When comparing LV MM values to 2D measurements, better agreement between short axis MM and 2D measurements has been found.^4^ When comparing the longitudinal and short axis view, Schober and Baade found that some measurements vary depending on the measuring plane, but this systematic difference was less than 5%, except for the interventricular septum in systole.^4^

When generating normal reference ranges for cardiac linear dimensions in dogs, the influence of body size on the echocardiographic measurements must be taken into account because of the wide range in the body size in dogs.^5^ Body weight (BW) is frequently used as a surrogate for body size and can be used in statistical regression models with a potentially nonlinear relationship between BW and the cardiac variables of interest. Based on such regression models, reference ranges can be derived as the prediction intervals (PIs) for specific BW values.

In veterinary medicine, so far, clinically usable BW‐dependent reference values for normal dogs have been established using ratio‐based indexing^6^ as well as allometric scaling (scaling to body mass)^5^; the latter being a regression approach. The currently commonly used reference ranges in dogs were generated using allometric scaling.^5^ Although these PIs generated are frequently used in clinical practice, our study had some limitations, the most important ones being that it only included 494 dogs, which, for this purpose, is a comparably small population, and more than 40% of the dogs were of a sighthound breed. Several studies have suggested that certain sighthound breeds have, compared to other breeds, different dimensional and functional cardiac variables.^7^, ^8^, ^9^, ^10^, ^11^, ^12^ In addition, there are many other single‐centered studies reporting of breed specific reference ranges in sighthound and nonsighthound breeds.^7^, ^8^, ^9^, ^10^, ^11^, ^12^, ^13^, ^14^, ^15^, ^16^, ^17^, ^18^, ^19^, ^20^, ^21^, ^22^, ^23^, ^24^, ^25^, ^26^, ^27^, ^28^, ^29^, ^30^, ^31^, ^32^, ^33^, ^34^, ^35^ Because the studies were single‐centered and because the findings were not compared to those of other breeds or a general dog population, it is not known if these specific ranges are truly deviant and if they are applicable in the multicenter environment, that is, have generalizability.

The aim of our study was to generate BW‐dependent PIs of the LV linear dimensions in a large population of dogs of many breeds in multicenter environment, and to identify breeds deviating from these intervals.

## 2 MATERIALS AND METHODS

### 2.1 Dogs

Data of healthy dogs of different breeds (Table S1) screened for breeding purposes from 2009 to 2016 were retrieved from the database of the Collegium Cardiologicum e.V. (CC). Examiners performing the echocardiographic studies were all certificated members of the CC. The examinations were conducted according to a standardized procedure and all results were recorded in an electronic protocol. Owner related data, signalment, auscultatory, ECG, and echocardiographic variables were retrieved.

### 2.2 Grouping of dogs

Dogs were grouped into 3 groups: All nonsighthound dogs, immaculate nonsighthound dogs, and breeds including sighthound dogs.

#### 2.2.1 All nonsighthound dogs

According to the classification system of the CC, dogs with no or minimal cardiovascular abnormalities are to be considered healthy. Dogs included in the all nonsighthound dog group had to meet the following criteria: Dogs had to be free of clinical signs of disease. They had to have none or, at the most, very mild insufficiencies of the atrioventricular or the semilunar valves, identified on the color‐Doppler echocardiogram. Dogs had to have normal anatomy of the ventricular outflow tracts, and had to have a physiological aortic and pulmonic flow velocity detected on the spectral‐Doppler echocardiogram, respectively (breed‐specific values were applied such as maximal aortic flow velocity of <2.4 m/s for Boxers or <2.0 m/s in Newfoundlands; flow velocities had to be within physiologic limits in dog breeds without any published maximal aortic and pulmonic flow velocities).

#### 2.2.2 Immaculate nonsighthound dogs

A subgroup of the *all nonsighthound dog* group consisted of dogs with no remarks on the echocardiogram. Dogs in this group had to meet the following requirements: Dogs must have been free of signs of disease and the physical examination did not reveal important or relevant abnormalities.

#### 2.2.3 Breeds including sighthound dogs

This group included dogs of sighthound breeds and dogs of nonsighthound breeds with >80 dogs. The dogs were considered to be healthy according to the classification system of the CC, as they, like the dogs in group *all nonsighthound dogs*, exhibited no or only minimal cardiovascular changes. Thus, they had to be free of clinical signs of disease and none or, at the most, mild insufficiencies of the atrioventricular or the semilunar valves were found on the color‐Doppler echocardiogram. Additionally, the anatomy of the ventricular outflow tracts had to be normal and the maximal aortic and pulmonic flow velocity had to be within physiologic limits, respectively.

### 2.3 Echocardiography

The echocardiographic examination was performed using a suitable ultrasound device, which provided the examiner with various Doppler techniques (spectral‐ and color‐Doppler) as well as simultaneous ECG recording. However, only the MM measurements were available for analysis within the scope of our study. The LV dimensions were measured either in the right parasternal long or short axis view.^36^ View of acquisition was noted in the report. The left ventricular septal and lateral wall thicknesses (interventricular septum in diastole and systole [IVSd and IVSs], respectively), left ventricular free wall in diastole and systole (LVWd and LVWs, respectively) and the left ventricular diameter were measured in systole and diastole (LVDd and LVDs, respectively). The diastolic measurements were performed at the beginning of the QRS complex; the systolic measurement was timed at the shortest distance between septum and lateral wall. The measurements were performed using the leading‐edge technique.^36^ The variable fractional shortening (FS)^37^ was then calculated automatically..^36^ The investigators were instructed to use breed‐specific values, if available. If no specific values had been published for the respective breed, the investigators were encouraged to interpret the results at one's own discretion, excluding all kinds of possible pathologic changes. Therefore, the PIs of Cornell et al^5^ were used as an aid to interpret results, but not as a definite criterion, because this would not take potential breed‐specific differences into account.

### 2.4 Statistical analysis

Groups analyzed separately included *all nonsighthound dogs* and *immaculate nonsighthound dogs*. The reference ranges were derived as PIs, which were estimated on the basis of statistical regression models. In the general outlier detection analysis, all variables were checked for possible outliers. In accordance with standard statistical practice, values outside the interval “upper/lower quartile ± triple interquartile range” were identified as outliers^38^ and then thoroughly inspected and excluded if necessary. This evaluation was performed separately for each breed where at least 50 dogs were available. Variables with skewed distributions were logarithmized in a preprocessing step to ensure a more symmetric distribution. In total, 197 measurements were removed from the analyses.

A simple linear regression model was estimated for each transformed variable, with the logarithmically transformed BW as the sole predictor.^39^ The estimated coefficients of the model, namely the model intercept and the weight effect, then constituted the variables *a* and *b* of the corresponding allometric scaling model, respectively. The appropriateness of these allometric scaling models was evaluated by estimating an additive regression model (GAM) for each variable, including a smooth, potentially nonlinear effect of BW to the power of the previously obtained variable *b*.^39^ Visual inspection of the linearity of the fitted prediction line was used to assess the adequacy of the allometric scaling model. This way we assured that the linear effect structure was a reasonable assumption for the observed relationship. Model estimation was performed based on the 2 data sets, the *all nonsighthound dogs* and the *immaculate nonsighthound dogs*. To compare these groups, the average difference between *all nonsighthound dogs* and *immaculate nonsighthound dogs* in their upper limits of the reference values across all variables were estimated. In order to do this, a GAM was used, including a dummy variable for the group and a nonlinear effect of BW.^39^ The latter was estimated based on a P‐spline basis, the model was fitted using the function gam from the package mgcv^39^ in the statistical software R.^40^

The PIs were generated in 2 differing ways: For variables that showed only a very weak association with BW, the values are presented as weight‐independent cut‐off values (5.0 percentile). Variables which were suitable for allometric scaling were subsequently fitted by a linear model using BW to the power of *b*. The PIs were then defined as the resulting 95% PIs based on this model. Additionally, estimates for approximate PIs were derived via Equation (1)^5^ from the allometric model. This method offers a simple way for on‐the‐spot calculations of PIs for different body weights

(1) $$\mathrm{PI}\;\text{borders}=\log^{-1}\;\left(\log\left(a\right)\pm \sigma \right)$$

The PIs calculated from these constants will approximate the PIs calculated directly from the linear model. The term log(*a*) is the intercept and *σ* is the square root of the estimated variance of the random error of the allometric model. Meanwhile *t* is the appropriate quantile of the Student's *t*‐distribution.

To determine whether certain breeds were within the generated PIs or not (deviant breeds), a cut‐off value of 10.0% above or below those PIs generated in our study was used. This means that a breed was identified as deviant breed if more than 10.0% of the measurements of dogs of this breed were above or below the corresponding PI.

Interobserver‐variability was analyzed using an additive mixed model for each variable, with the examiner effect as random intercept and a nonlinear effect of BW. The model was estimated only based on the breed of Boxers (from the group *breeds including sighthound dogs*), since they constitute the largest group within the sample and to prevent biased results caused by comparing different breeds. The proportion of the error variance (residual variance [RVa]) explained by the examiner effect was then used as measure for interobserver‐variability.^41^ The analyses were performed using the statistical software R 3.5.0.^40^ For model estimation, the package mgcv v1.8‐24 was used.^39^

## 3 RESULTS

### 3.1 Dogs

A total of 48 examiners had conducted the echocardiographic examinations. The group *all nonsighthound dogs* included 6097 dogs. Of these 6097 dogs, 56.8% were male and 43.2% female. The median BW was 29.3 kg (IQR, 25.0‐37.7 kg) and the mean age was 2.6 years. A total of 1794 dogs met the inclusion criteria of the group *immaculate nonsighthound dogs*. Of these animals, 42.3% were male and 57.7% female. The median BW was 30.0 kg (IQR, 24.7 kg‐45.0 kg) and the mean age of these dogs was 2.2 years.

The following 13 breeds were represented by more than 80 dogs in the data set: Afghans (n *=* 306), Boxers (n *=* 3111), Cavalier King Charles Spaniels (n *=* 94), Doberman Pinschers (n *=* 427), French Bulldogs (n *=* 203), Golden Retrievers (n *=* 89), Great Danes (n *=* 900), Hovawarts (n *=* 184), Irish Wolfhounds (n *=* 837), Labrador Retrievers (n *=* 159), Newfoundlands (n *=* 161), Polski Owczarek Nizinnys (n *=* 121), and Salukis (n *=* 302). Of these 13 breeds, 3 were sighthound breeds (Afghans, Irish Wolfhounds, and Salukis). In addition to these 3 sighthound breeds, the following 9 sighthound breeds were also represented in the data set: Borsoi (n *=* 9), Deerhound (n *=* 7), Italian Greyhound (n *=* 13), Galgo Espanol (n *=* 2), Greyhound (n *=* 2), Magyar Agar (n *=* 1), Silken Windsprite (n *=* 25), Sloughi (n *=* 3), and Whippet (n *=* 47). Descriptive data of the breeds are listed in the Table S2.

### 3.2 Comparison of groups and generation of weight‐dependent 95% PIs

We observed a strong association between BW and all dimensional cardiac variables, which were accurately described by allometric scaling. No such association was found for FS; in the group *all nonsighthound dogs*, the *R*^2^ for FS was 0.040, and in the group *immaculate nonsighthound dogs* the *R*^2^ was 0.041. However, FS showed a weak inverse correlation to BW as it decreased minimally with increasing BW (*b* was −0.065). The calculated cut‐off value (5.0 percentile) was 23.36. The allometric scaling formulas of the echocardiographic measurements for the *all nonsighthound dogs* and *immaculate nonsighthound dogs* are presented in Table 1. Comparing populations using a GAM model, which described the mean course of the respective upper limits of the reference ranges, showed a minimal difference between the 2 populations for all studied echocardiographic variables (Figure S1). Furthermore, no relevant differences between the groups could be detected when the mean differences between the upper limits of the *all nonsighthound dogs* and *immaculate nonsighthound dogs* were inspected (Table S3). Hence, further analyses were conducted using the group *all nonsighthound dogs* only. Consequently, constants for the indexation of the echocardiographic measurements (Table 2) as well as 95% PIs (Table 3) were presented for each variable using respective group.

**TABLE 1:** Results of regression analysis using allometric scaling to body weight (*Y* = *a* BW^*b*^) in 6097 dogs of the all nonsighthound group and 1794 dogs of the immaculate nonsighthound group

Variable	All nonsighthound dogs	*R* ^2^	Immaculate nonsighthound dogs	*R* ^2^
LVDd	1.38 × BW^0.322^	0.766	1.32 × BW^0.335^	0.887
LVDs	0.87 × BW^0.346^	0.680	0.79 × BW^0.370^	0.827
IVSd	0.36 × BW^0.289^	0.494	0.35 × BW^0.299^	0.684
IVSs	0.51 × BW^0.276^	0.484	0.50 × BW^0.287^	0.661
LVWd	0.40 × BW^0.261^	0.465	0.37 × BW^0.278^	0.607
LVWs	0.60 × BW^0.247^	0.487	0.57 × BW^0.259^	0.634

Abbreviations: IVSd, interventricular septum in diastole; IVSs, interventricular septum in systole; LVDd, left ventricular diameter in diastole; LVDs, left ventricular diameter in systole; LVWd, left ventricular free wall in diastole; LVWs, left ventricular free wall in systole.

**TABLE 2:** Constants for indexing the M‐mode measurements as well as the scaling exponents from the allometric models that allow the approximate construction of the prediction intervals of the group *all nonsighthound dogs* (n = 6097)

Variable	97.5 Percentile	95.0 Percentile	75.0 Percentile	50.0 Percentile	25.0 Percentile	5.0 Percentile	2.5 Percentile	Exponent
LVDd	1.63	1.59	1.46	1.38	1.30	1.20	1.17	0.322
LVDs	1.09	1.05	0.94	0.87	0.81	0.72	0.70	0.346
IVSd	0.49	0.47	0.40	0.36	0.33	0.28	0.27	0.289
IVSs	0.68	0.65	0.56	0.51	0.46	0.40	0.38	0.276
LVWd	0.53	0.51	0.44	0.40	0.36	0.31	0.30	0.261
LVWs	0.78	0.75	0.65	0.60	0.55	0.48	0.46	0.247

Abbreviations: IVSd, interventricular septum in diastole; IVSs, interventricular septum in systole; LVDd, left ventricular diameter in diastole; LVDs, left ventricular diameter in systole; LVWd, left ventricular free wall in diastole; LVWs, left ventricular free wall in systole.

**TABLE 3:** Mean value and 95% prediction intervals (in centimeters) of the group *all nonsighthound dogs* (n = 6097)

BW (kg)	LVDd	LVDs	IVSd	IVSs	LVWd	LVWs
2	1.7 (1.5‐2.0)	1.1 (0.9‐1.4)	0.4 (0.3‐0.6)	0.6 (0.5‐0.8)	0.5 (0.4‐0.6)	0.7 (0.5‐0.9)
3.5	2.1 (1.7‐2.4)	1.3 (1.1‐1.7)	0.5 (0.4‐0.7)	0.7 (0.5‐1.0)	0.6 (0.4‐0.7)	0.8 (0.6‐1.1)
5	2.3 (2.0‐2.7)	1.5 (1.2‐1.9)	0.6 (0.4‐0.8)	0.8 (0.6‐1.1)	0.6 (0.4‐0.8)	0.9 (0.7‐1.2)
7.5	2.6 (2.2‐3.1)	1.8 (1.4‐2.2)	0.6 (0.5‐0.9)	0.9 (0.7‐1.2)	0.7 (0.5‐0.9)	1.0 (0.8‐1.3)
10	2.9 (2.4‐3.4)	1.9 (1.5‐2.4)	0.7 (0.5‐1.0)	1.0 (0.7‐1.3)	0.7 (0.5‐1.0)	1.1 (0.8‐1.4)
12.5	3.1 (2.6‐3.7)	2.1 (1.7‐2.6)	0.8 (0.6‐1.0)	1.0 (0.8‐1.4)	0.8 (0.6‐1.0)	1.1 (0.9‐1.5)
15	3.3 (2.8‐3.9)	2.2 (1.8‐2.8)	0.8 (0.6‐1.1)	1.1 (0.8‐1.4)	0.8 (0.6‐1.1)	1.2 (0.9‐1.5)
17.5	3.5 (2.9‐4.1)	2.3 (1.9‐2.9)	0.8 (0.6‐1.1)	1.1 (0.8‐1.5)	0.8 (0.6‐1.1)	1.2 (0.9‐1.6)
20	3.6 (3.1‐4.3)	2.5 (2.0‐3.1)	0.9 (0.6‐1.2)	1.2 (0.9‐1.6)	0.9 (0.6‐1.2)	1.3 (1.0‐1.6)
22.5	3.8 (3.2‐4.4)	2.6 (2.0‐3.2)	0.9 (0.7‐1.2)	1.2 (0.9‐1.6)	0.9 (0.7‐1.2)	1.3 (1.0‐1.7)
25	3.9 (3.3‐4.6)	2.7 (2.1‐3.3)	0.9 (0.7‐1.2)	1.2 (0.9‐1.7)	0.9 (0.7‐1.2)	1.3 (1.0‐1.7)
27.5	4.0 (3.4‐4.7)	2.7 (2.2‐3.4)	0.9 (0.7‐1.3)	1.3 (0.9‐1.7)	0.9 (0.7‐1.3)	1.4 (1.0‐1.8)
30	4.1 (3.5‐4.9)	2.8 (2.3‐3.5)	1.0 (0.7‐1.3)	1.3 (1.0‐1.7)	1.0 (0.7‐1.3)	1.4 (1.1‐1.8)
32.5	4.2 (3.6‐5.0)	2.9 (2.3‐3.6)	1,0 (0,7‐1,3)	1.3 (1.0‐1.8)	1.0 (0.7‐1.3)	1,4 (1,1‐1,8)
35	4.3 (3.7‐5.1)	3.0 (2.4‐3.7)	1.0 (0.7‐1.4)	1.4 (1.0‐1.8)	1.0 (0.7‐1.4)	1.4 (1.1‐1.9)
40	4.5 (3.8‐5.3)	3.1 (2.5‐3.9)	1.0 (0.8‐1.4)	1.4 (1.0‐1.9)	1.0 (0.8‐1.4)	1.5 (1.1‐1.9)
45	4.7 (4.0‐5.6)	3.3 (2.6‐4.1)	1.1 (0.8‐1.5)	1.5 (1.1‐2.0)	1.1 (0.8‐1.4)	1.5 (1.2‐2.0)
50	4.9 (4.1‐5.7)	3.4 (2.7‐4.2)	1.1 (0.8‐1.5)	1.5 (1.1‐2.0)	1.1 (0.8‐1.5)	1.6 (1.2‐2.0)
55	5.0 (4.2‐5.9)	3.5 (2.8‐4.4)	1.2 (0.8‐1.6)	1.5 (1.1‐2.1)	1.1 (0.8‐1.5)	1.6 (1.2‐2.1)
60	5.2 (4.4‐6.1)	3.6 (2.9‐4.5)	1.2 (0.9‐1.6)	1.6 (1.1‐2.1)	1.2 (0.9‐1.6)	1.6 (1.3‐2.1)
65	5.3 (4.5‐6.3)	3.7 (3.0‐4.6)	1.2 (0.9‐1.6)	1.6 (1.2‐2.2)	1.2 (0.9‐1.6)	1.7 (1.3‐2.2)
70	5.4 (4.6‐6.4)	3.8 (3.0‐4.7)	1.2 (0.9‐1.7)	1.6 (1.2‐2.2)	1.2 (0.9‐1.6)	1.7 (1.3‐2.2)
75	5.5 (4.7‐6.5)	3.9 (3.1‐4.9)	1.3 (0.9‐1.7)	1.7 (1.2‐2.3)	1.2 (0.9‐1.7)	1.7 (1.3‐2.3)

Abbreviations: BW, body weight; IVSd, interventricular septum in diastole; IVSs, interventricular septum in systole; LVDd, left ventricular diameter in diastole; LVDs, left ventricular diameter in systole; LVWd, left ventricular free wall in diastole; LVWs, left ventricular free wall in systole.

[Correction added on October 22, 2020 after first online publication: Table 3 “Mean value and 95% prediction intervals (in centimeters) of the group all non‐sighthound dogs (n = 6097)” LVIDd, LVDs, IVSd, IVSs, LVWd and LVWs values corrected.]

### 3.3 Analysis of breeds including sighthound dogs

**FIGURE 1:**
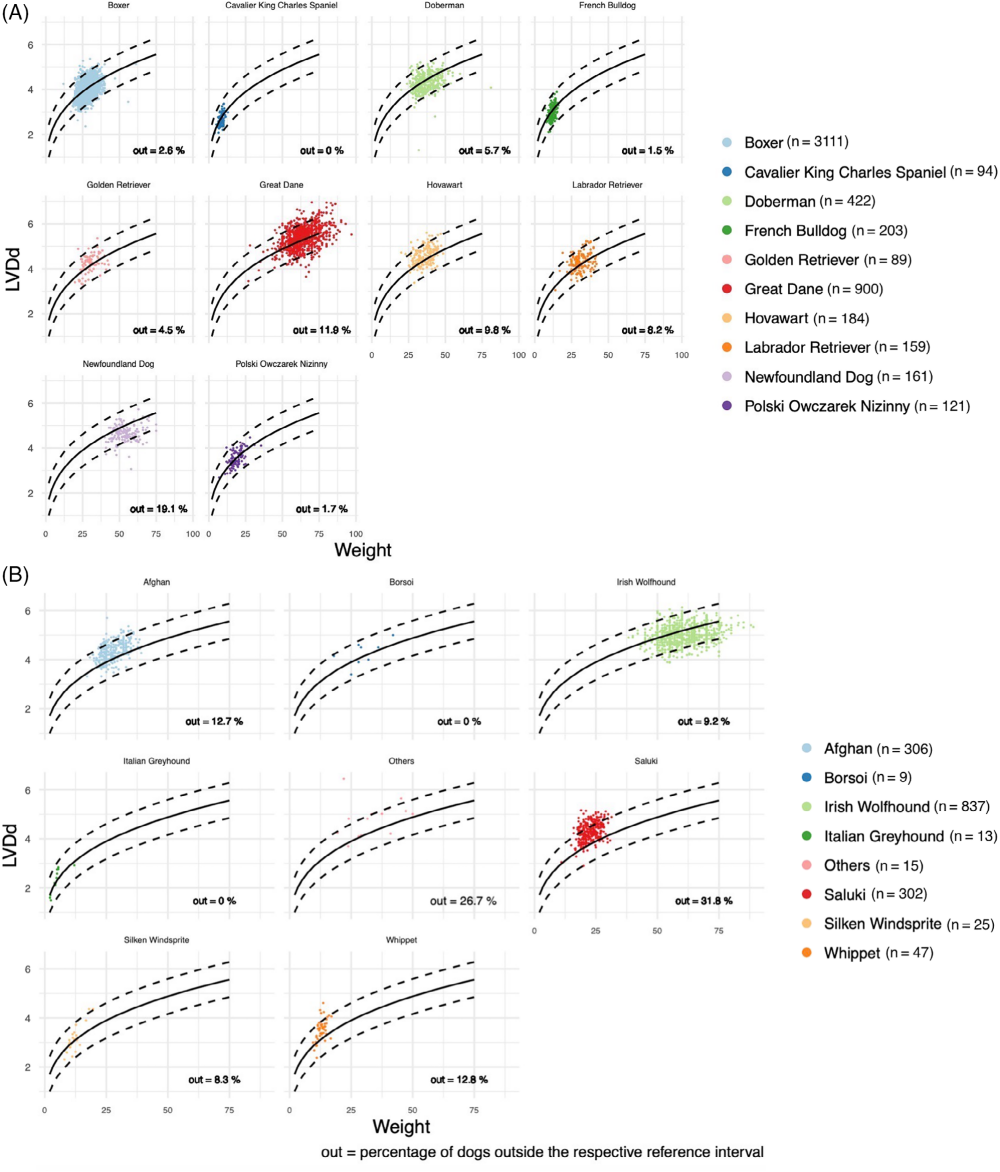
Scatter plots with superimposed regression lines (solid lines) and 95% prediction intervals (broken lines) by breed for the LVDd as a function of body weight for breeds in the nonsighthound breed group (A) and for breeds in the sighthound group (B). The plots show how observed values fit inside the 95% PIs. LVDd, left ventricular diameter in diastole; PI, prediction interval

The results of the analyses regarding the breed distribution (percentage above and below the respective PIs) are summarized in Table 4. As already described, a cut‐off value of 10.0% above or below the PIs was used to identify deviant breeds. Of the 10 nonsighthound breeds with >80 dogs, all measurements of the breeds Boxer, Cavalier King Charles Spaniel, Doberman Pinscher, French Bulldog, Labrador Retriever, and Polski Owczarek Nizinny had less than 10% of the observations above or below the PIs generated. In each of the breeds, Golden Retriever and Hovawart 1 variable could be detected where more than 10.0% of the measurements were above or below the corresponding PI; in the Golden Retriever, 14.1% of the measurements were above the PI of LVWs and in the Hovawart, 10.4% of the measurements were above the PI of LVDs. In contrast, in Newfoundlands, more than 10.0% of the measurements were below the generated PIs for several variables. Figure 1A shows the results of the analysis for the LVDd dimension of nonsighthounds.

**TABLE 4:** Percentage of dogs in each breed outside (above or below) the 95% prediction intervals generated in the *breeds including sighthounds group* (n = 7003)

	N	LVDd		LVDs		IVSd		IVSs		LVWd		LVWs	
		↑%	↓%	↑%	↓%	↑%	↓%	↑%	↓%	↑%	↓%	↑%	↓%
Afghan*	306	12.7	—	11.8	0.3	5.6	1.0	5.6	1.0	4.4	1.0	4.6	1.3
Barsoi*	9	—	—	11.1	—	—	—	—	—	—	—	—	11.1
Boxer	3111	1.5	1.1	1.0	1.8	1.5	1.9	1.8	1.2	1.9	1.7	2.5	1.0
Cavalier King Charles Spaniel	94	—	—	—	1.1	—	—	—	—	—	—	—	1.1
Doberman	427	2.6	3.1	2.6	1.7	2.1	3.1	1.4	2.1	3.1	4.1	2.1	2.9
French Bulldog	203	—	1.5	—	3.0	0.5	—	1.5	—	0.5	0.5	2.0	—
Golden Retriever	89	3.4	1.1	5.7	4.5	3.5	—	7.0	—	4.8	2.3	14.1	1.2
Great Dane	900	8.6	3.3	8.7	3.8	8.0	2.5	9.9	3.1	6.1	2.5	7.3	3.9
Hovawart	184	9.8	—	10.4	1.1	8.2	0.6	3.8	1.1	8.4	0.5	7.8	1.1
Italian Greyhound*	13	—	—	—	—	—	—	—	—	—	—	—	—
Irish Wolfhound*	837	1.7	7.5	1.0	7.4	1.6	7.5	1.4	12.0	2.3	7.7	2.5	8.6
Labrador Retriever	159	7.5	0.7	6.3	2.5	1.3	0.6	3.1	1.3	—	3.5	1.3	1.3
Newfoundland Dog	161	—	19.1	—	14.0	1.9	7.6	1.2	7.1	1.3	10.9	1.9	14.0
Polski Owczarek Nizinny	121	1.7	—	3.3	0.8	0.8	0.8	0.8	0.8	—	1.7	1.7	0.8
Saluki*	302	31.5	0.3	28.5	—	9.3	0.3	7.0	—	4.8	1.0	7.7	—
Silken Windsprite*	25	8.3	—	—	4.2	—	—	4.2	—	4.2	—	—	—
Whippet*	47	12.8	—	17.0	—	2.1	—	8.5	—	2.1	—	—	—
Other Sighthound Breeds*	15	26.7	—	33.3	—	33.3	—	20.0	—	20.0	—	6.7	—

*Note*: Sighthound breeds are marked with an asterisk. N = number of dogs, ↑% = percentage of measurements above the generated prediction intervals of the respective breed, ↓% = percentage of measurements below the generated prediction intervals of the respective breed.

Abbreviations: IVSd, interventricular septum in diastole; IVSs, interventricular septum in systole; LVDd, left ventricular diameter in diastole; LVDs, left ventricular diameter in systole; LVWd, left ventricular free wall in diastole; LVWs, left ventricular free wall in systole.

Regarding the sighthound breeds, the following could be observed: In the Afghan, Saluki, and the group other sighthound breeds (Deerhound, Galgo Espanol, Greyhound, Magyar Agar and Sloughi), a high percentage of measurements in several dimensions could be detected above the PIs. In the Afghan, Saluki, and Whippet, more than 10.0% of the measurements of the variables LVDd (12.7%, 31.5%, and 12.8%, respectively) and LVDs (11.8%, 28.5%, and 17.0%, respectively) were above the respective PIs and in the group of other sighthound breeds a percentage of more than 20% above the respective PIs could be detected for all variables (except LVWs).

In the Borsoi breed, however, only for LVDs a high percentage of observations (11.1%) was found to be above the upper limits of the PIs. In the cases of the Italian Greyhound and the Silken Windsprite, all measurements were less than 10% above or below the PIs.

In contrast, for Irish Wolfhound, none of the variables had a high percentage (>10.0%) of measurements above the PIs, and only 1 variable had a high percentage of observations below the PI (IVSs, 12.0%). Figure 1B shows the results of the analysis for the LVDd dimension of nonsighthounds in graphical form.

### 3.4 Interobserver‐variability

The maximal interobserver‐variability, thus the maximal values of RVa explained by the examiner effect, could be detected for the variable LVWd with 30.6%. The variances of the other measurements ranged between 8.1% and 24.0%, with the lowest values for LVDs and LVDd. In the Table S4, all measurements with corresponding variances are listed.

## 4 DISCUSSION

Studying a presumably healthy sample of a population is the most commonly utilized statistical approach to establishing reference intervals. In veterinary medicine, this approach is recommended in the guidelines of the Quality Assurance and Laboratory Standards committee of the American Society for Veterinary Clinical Pathology and should lead to the generation of comparable and carefully thought‐out reference ranges.^42^ However, this procedure has the disadvantage of misclassifying some healthy individuals as abnormal due to physiologic variations.^43^ If, for example, reference ranges were calculated using dogs considered to be normal applying Cornell's PIs, the reference range would be diminished by 2.5% at each end and a larger number of dogs would incorrectly be categorized as abnormal. Because the examiners performing echocardiography in our study were encouraged to interpret the echocardiographic results at their own discretion (especially if no breed‐specific values were available), and systematically exclude all types of cardiac abnormalities, the above described problem was avoided.

In addition, to generate widely applicable echocardiographic reference ranges for cardiac linear dimensions in dogs, the influence of body size must be taken into account.^5^ Several studies in dogs have shown that the body size or the BW have a clinically relevant impact on most dimensional echocardiographic variables.^5^, ^7^, ^8^, ^9^, ^18^, ^20^, ^25^, ^26^, ^33^, ^44^, ^45^, ^46^, ^47^, ^48^ However, most of these studies were single centered. Thus, when transferring the results of these studies to the general dog population, their applicability might be limited. In contrast, our study represents a multicentered environment, which consequently leads to a high generalizability.

Furthermore, not only BW, but also the somatotype (eg, breeds of different sizes but uniform athletic physiques, like sighthounds) and breed have to be considered when interpreting echocardiograms. Sighthounds are known to exhibit several peculiarities in the dimensions and functional variables of the heart. For example, Greyhounds have, compared to other dogs, both larger LV dimensions and increased wall thickness of the IVS and LVW.^7^, ^9^, ^10^, ^11^ Comparable results have been reported in Whippets,^7^, ^8^, ^12^ as well as in Deerhounds.^22^ The exact reason for these findings has not yet conclusively been determined, but selection for athletic^7^ capacity and increased blood viscosity^11^ have been suggested as possible causes. Thus, to take these characteristics of sighthounds into account, we removed the sighthounds from the study population (*all nonsighthound dogs*) to generate the BW depended PIs.

In our study, we found a strong association between the logarithmically transformed echocardiographic measurement and BW for all cardiac dimensions. Association was the strongest for LV chamber dimensions in diastole and systole (*R*^2^ = 0.766 and 0.680, respectively). If allometric scaling is used to normalize cardiac dimensions to BW, volumes should theoretically linearly relate to BW, cross‐sectional areas should be proportional to BW^2^ ^/3^, and linear dimensions should linearly relate to BW^1/3^.^5^ As cardiac chamber diameters and wall thicknesses are 1‐dimensional measured variables (straight lines), the scaling exponent of the allometric formulas should theoretically approximate one‐third. In our study, the exponents of all cardiac dimensions approximated this theoretical value of one‐third and ranged between 0.247 and 0.346 (Table 2). Furthermore, it was found that the cardiac wall thicknesses had lower values for the scaling exponents (constant *b*) compared to the other 2 measurements—an observation which is consistent with the results of the publication of Cornell et al.^5^

**FIGURE 2:**
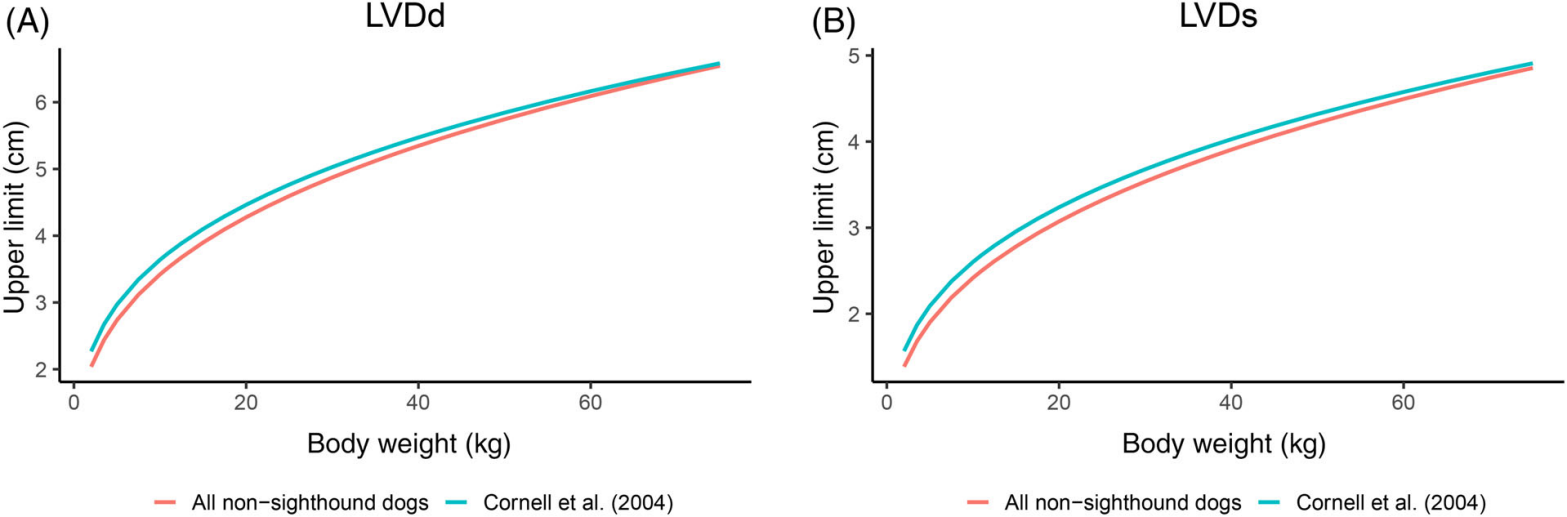
Upper limits of 95% PIs of, A, LVDd and, B, LVDs as a function of body weight in the all nonsighthound group generated using a GAM (red line, n = 6097) and predicted values using the formulas provided in the study by Cornell et al^5^ (blue line, n = 494). The upper limit of the 95% PI was slightly lower in our study compared to the upper PI limit provided by Cornell et al. GAM, generalized additive model; LVDd, left ventricular diameter in diastole; LVDs, left ventricular diameter in systole; PI, prediction interval [Correction added on October 22, 2020 after first online publication: Figure 2 “Upper limits of 95% PIs of, A, LVDd and, B, LVDs as a function of body weight in the all non‐sighthound group generated using a Q7 GAM (red line, n = 6097) and predicted values using the formulas provided in the study by Cornell et al5 (blue line, n = 494)” LVDd and LVDs upper PI limit graph lines corrected.]

When comparing the generated PIs of our study with those currently provided by Cornell et al, both differences and similarities were found.^5^ The estimates for allometric constants and PIs for cardiac wall thicknesses were very similar between the 2 studies, but notable differences could be observed for the LV dimensions. These differences were also evident in the graphical representation of the mean course of the respective upper limits of the PIs. In the graphical representation, the upper limits of PIs generated in our study were almost exclusively below the upper limits of Cornell et al^5^; exceptions were IVSs and LVWd below 10.0 kg and below 15.0 kg, respectively. Figure 2 shows the graphical representation for the variable LVDd. The differences between the upper limits of LV dimensions could be explained by the exclusion of sighthounds in our study population. The comparably large proportion (over 40%) of sighthound breeds included in the study population of Cornell et al, presumably have had an influence on the reference values as well as the constants for the indexation of this MM measurement generated.^5^

The constants for indexing echocardiographic measurements allow, in combination with the respective exponents, the (approximate) calculation of PIs as a function of BW and are important for identifying outlier measurements. Because our study excluded sighthound breeds when generating the constants for the allometric formulas and PIs, the effect of BW on LV measurements was slightly lower compared to those described in the Cornell et al study.^5^ For example, the 2.5 percentile for the constant for the variable LVDd was 1.27 and the 97.5 percentile was 1.85 in the Cornell et al study.^5^ For a dog weighing 25.0 kg, the reference range for the variable LVDd basis of the constants from the Cornell et al^5^ study is 1.27 × 25.0^0.294^ = 3.3 cm to 1.85 × 25.0^0.294^ = 4.8 cm. If the constants of our study were used, then the 2.5 percentile of the constant *a* is 1.17 and the 97.5 percentile is 1.63 for the LVDd. The reference range for a 25.0 kg dog using the constants and the exponent (0.322) in our study was between 1.17 × 25.0^0.322^ = 3.3 cm and 1.63 × 25.0^0.322^ = 4.6 cm. Consequently, a 25.0 kg dog with a LVDd of 4.7 cm would be within the reference range using the Cornell et al reference ranges,^5^ but above upper PI using the results of our study.

The results of our study also have an impact on PIs in smaller dogs. For example, in the “EPIC study”,^49^ 1 of the inclusion criteria and indicators for cardiomegaly was a normalized LVDd of ≥1.7, which is approximately equivalent to the 95.0 percentile of the population in the Cornell et al study.^5^ If, for example, the EPIC inclusion criteria are applied to a 12.0 kg dog, then the LVDd must be equal or greater than 1.70 × 12.0^0.294^ = 3.5 cm. If the estimates of our study are applied, then the upper 95% PI is 1.63 × 12.0^0.322^ = 3.6 cm. By comparison, the PI using the Cornell et al formula is 1.85 × 12.0^0.294^ = 3.8 cm.^5^ This observation underlines the quality of the PIs generated in our study.

An advantage of our study was that the validity of the calculated PIs was confirmed by validating the generated values via the GAM. The GAM is a nonparametric model, which can be used to validate other statistical models (such as allometric models) for accuracy. In contrast to “model‐driven” methods, which only allow for a response curve limited by the underlying statistical method, the GAM is “data‐driven” and actually represents the functional response curve of the data.^39^ The degree of fitness for other models can therefore be validated by analyzing the deviance. Regarding our analysis, the better fit between observations and the linear the regression line of the GAM, the better the fitness of the examined allometric model. The degree of fit of the GAM was adequate for the allometric models of all dimensions in the group *all nonsighthound dogs*, which further supports our results.

With regard to the breed distribution, the following could be seen: For most breeds, the measurements of the nonsighthounds were largely within the generated PIs, with the exception of Newfoundlands, where a significant number of measurements were below the PIs. In the case of the sighthounds, the measurements were above the generated PIs for most breeds, especially in the LV chamber dimensions. As an exception to this, the Irish Wolfhound could be detected, where the measurements were in higher percentages below the generated PIs.

Looking at the results of the breed analysis in detail, it was found that (almost) all measurements of the nonsighthound breeds Boxer, Cavalier King Charles Spaniel, French Bulldog, Doberman Pinscher, Golden Retriever, Great Dane, Hovawart, Labrador Retriever, and Polski Owczarek Nizinny were either within the generated PIs or at least within the defined limits for “nondeviant breeds” (a breed was identified as deviant breed if more than 10.0% of the measurements of dogs of this breed were above or below the corresponding PI).

It can therefore be concluded that, in principle, the generally applicable PIs can be used for these breeds, although breed specific MM values, if available, might be preferred. In addition, it must be taken into account that in both, Great Danes and Hovawarts, some of the measurements were only slightly below the 10.0% limit. In the Great Dane, the variables LVDd (8.6%), LVDs (8.7%), IVSd (8.0%), and IVSs (9.9%) stand out, and in the Hovawart, in addition to LVDs (10.4%), LVDd (9.8%), IVSd (8.2%), and LVWd (8.4%) also stand out. Therefore, although only 1 variable in each of these breeds had more than 10.0% of measurements above the corresponding PI, breed specific values for the Great Dane^24^ and Howavart might be indicated. A high percentage of measurements in almost all dimensions in the Newfoundlands was clearly outside the generated PIs,^23^ which suggests that breed specific PIs are also indicated in this breed.

Concerning the sighthound breeds, our study showed that in the Afghan, the Saluki, the Whippet, and the other sighthound breeds, the measurements of several dimensions—especially the LV chamber dimensions—were clearly above the cut‐off limit of 10.0%. Therefore, as generally assumed for sighthound breeds, higher values of the LV dimensions can be presumed for these breeds.

With the Irish Wolfhound, which had a large number of measurements (n = 837), an interesting finding was observed. In all dimensions, at least 7.4% (and up to 12.0%) of the measurements were below the generated PIs. This suggests that the Irish Wolfhound is different from other sighthound breeds, such as the Afghan or the Saluki. Therefore, breed specific PIs^30^ are indicated in this breed as well.

In contrast, the situation is less clear for the Borsoi, the Italian Greyhound, and the Silken Windsprite. Although for the Borsoi, 11.1% of the measurements were above the PI for LVDs, also 11.1% of the measurements for LWVs were below the respective PI. Furthermore, for the Italian Greyhound and the Silken Windsprite, all measurements were even within the generated PIs. This could be due to the low numbers of animals in these breeds (Borsoi, n = 9; Italian Greyhound, n = 13; Silken Windsprite, n = 25); on the other hand, however, it must also be considered that the assumption that the cardinal dimensions of all sighthound breeds are above the general dog population cannot be applied in principle. Therefore, these 3 breeds in particular should be investigated more intensively in follow‐up studies with larger study populations.

## 5 LIMITATIONS

Most of the limitations of this article relate to its retrospective nature. The data used were generated over a period of 7 years by many investigators with different experience and by means of different types of ultrasound equipment. Especially due to these heterogeneous individual and technical aspects, a certain degree of variability cannot be avoided. However, it must also be stressed that this heterogeneity also leads to a higher generalizability, as all causes of variability were included, and this is a clear advantage of our study.

In addition, the standardized examination protocol of the CCs contained a certain degree of freedom of decision for the examiner. For example, the LV measurements could be performed in either the longitudinal or in the short axis,^36^ although a study from the year 2000 suggested that the sectional plane used for acquisition has an effect on LV measurements.^4^

Due to a relatively high number of postanalytical transmission errors such as typos or missing data, fewer animals were available for the analyses of the *immaculate nonsighthound dogs* group.

In addition, the breeds Boxer and Great Dane were overrepresented. Furthermore, there were only a few dogs with a BW under 10.0 kg in the data pool. This could have affected the accuracy of the PIs in lower weight categories. Regarding the breeds, especially the sighthound breeds, some had only a few dogs included, and these breeds should be further evaluated in larger studies with more dogs.

## 6 CONCLUSIONS

Generally applicable BW‐dependent PIs were generated for the LV linear dimensions in a large population of dogs. Findings of our study reinforce that these PIs are valid for most nonsighthound dogs, except the breed Newfoundland, where breed specific PIs are indicated. However, breed specific PIs might also be indicated in the Great Dane and the Hovawart. With regard to the sighthounds, it can be concluded that specific PIs are indicated in these breeds as most of the sighthound breeds deviated strongly from the generated PIs.

## CONFLICT OF INTEREST DECLARATION

Authors declare no conflict of interest.

## OFF‐LABEL ANTIMICROBIAL DECLARATION

Authors declare no off‐label use of antimicrobials.

## INSTITUTIONAL ANIMAL CARE AND USE COMMITTEE (IACUC) OR OTHER APPROVAL DECLARATION

Authors declare no IACUC or other approval was needed.

## HUMAN ETHICS APPROVAL DECLARATION

Authors declare human ethics approval was not needed for this study.

## Supporting information


**Supplementary Figure 1** The upper limits of 95% prediction intervals (PI) of LVDd (A) and LVDs (B) as a function of body weight. The curves were generated by a GAM model of the 2 populations all nonsighthound dogs (red line, n = 6097) and immaculate nonsighthound dogs (green line, n = 1794).

**Supplementary Table 1** Number of dogs in each breed in all 7651 dogs (including sighthounds).
**Supplementary Table 2**: Characteristics of dogs of breeds (n = 7003) in which echocardiographic measurements were compared to the 95% prediction intervals generated in the all nonsighthound group (n = 6097). Sighthound breeds are marked with an asterisk. N = Number of dogs, BW = Median body weight, Age = Mean age.
**Supplementary Table 3**: Estimates of mean difference between the upper limits (of all measured variables LVDd, LVDs, IVSd, IVSs, LVWd and LVWs) of the groups all nonsighthound dogs (n = 6097) and immaculate nonsighthound dogs (n = 1794) based on a generalized additive model including a nonlinear effect of body weight.
**Supplementary Table 4**: Estimated interobserver variability (in percent) based on the weight‐independent residual variance for the examined echocardiographic variables in the all nonsighthound group (n = 6097)
