# Supplementary figures and images for: Left ventricular M‐mode prediction intervals in 7651 dogs: Population‐wide and selected breed‐specific values
Source: J Vet Intern Med. 2020 Oct 2;34(6):2242–52. doi: 10.1111/jvim.15914 (PMC7694859; doi:10.1111/jvim.15914)

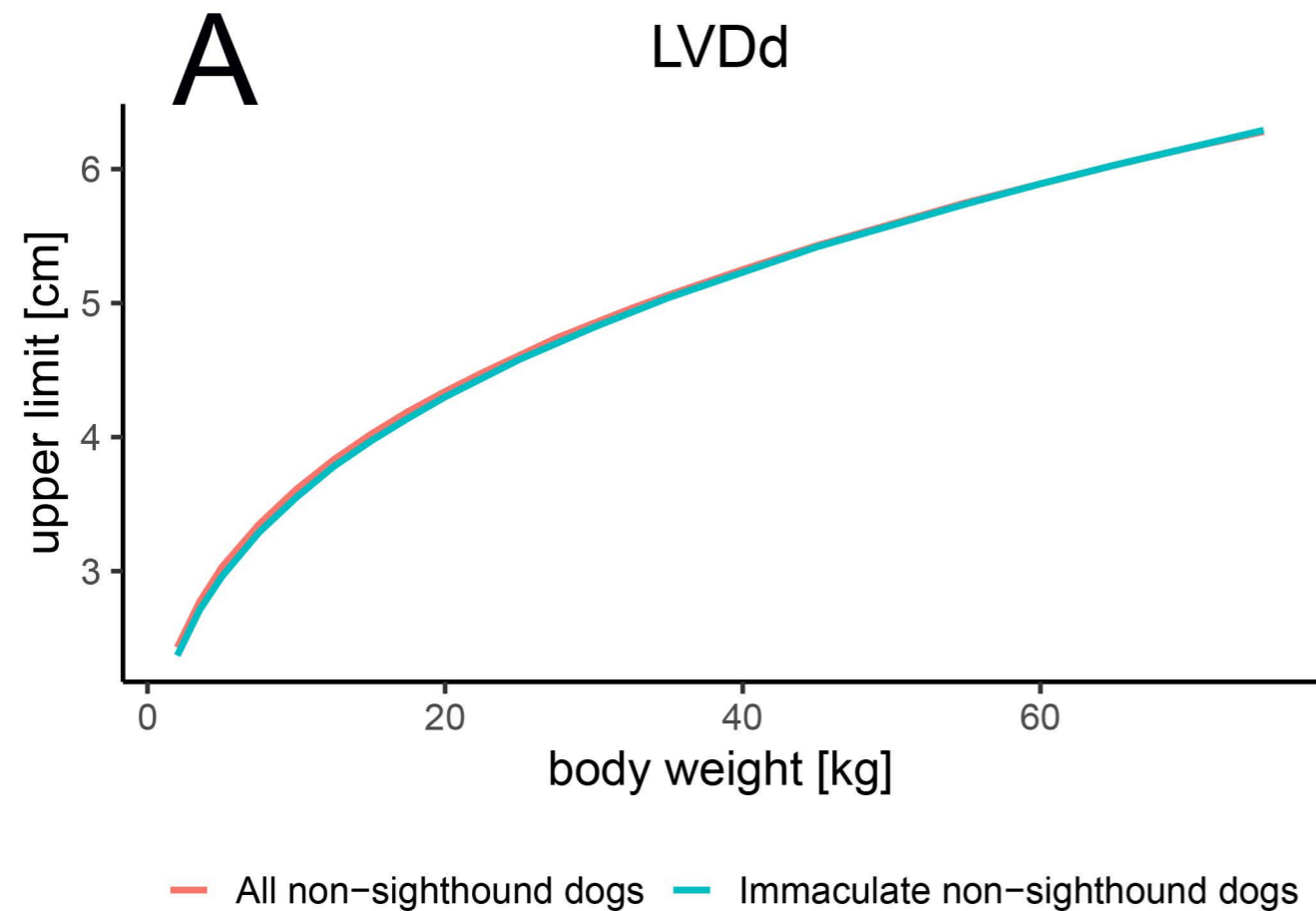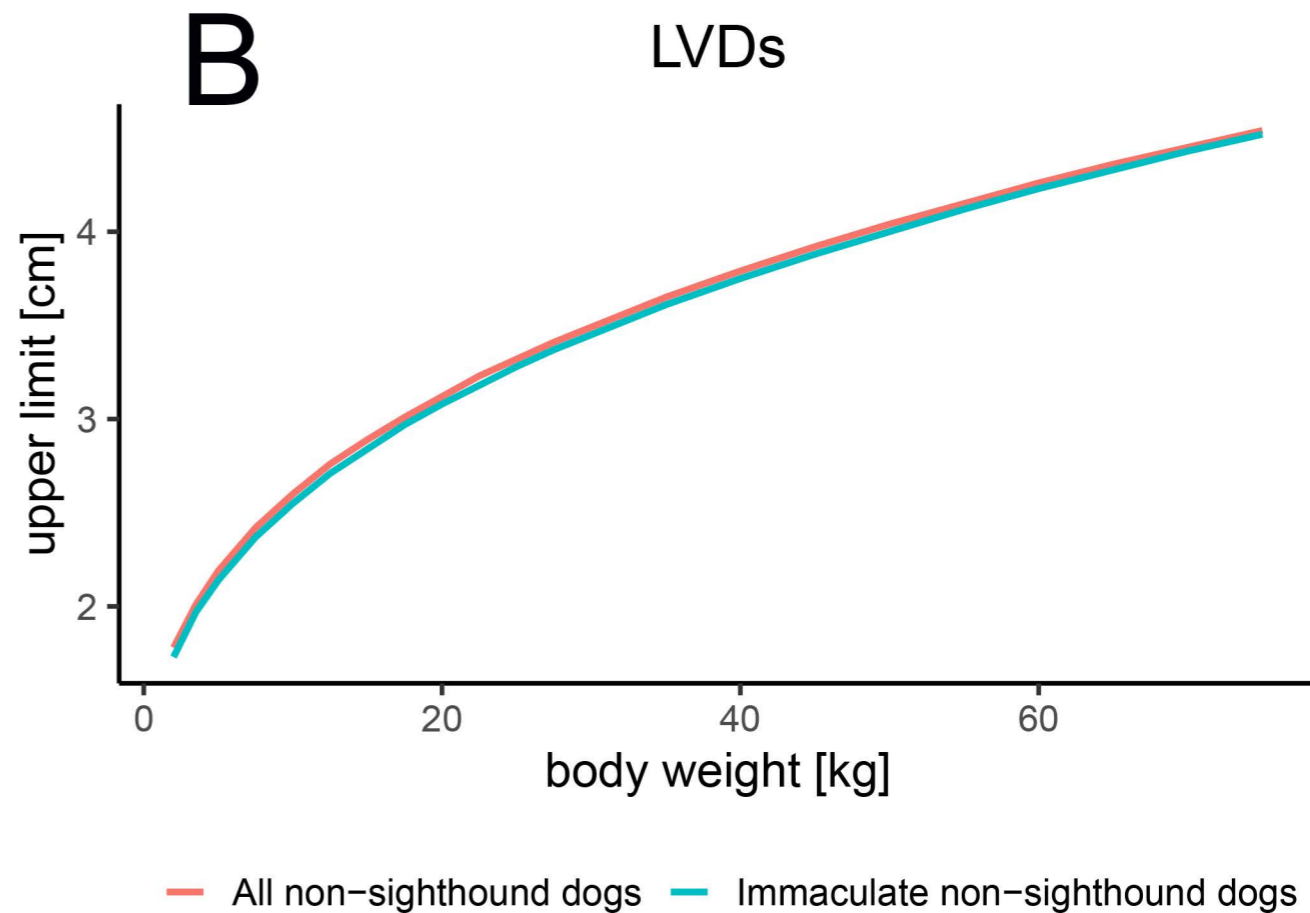

Supplement: Supplementary file 1 — Supplementary Figure 1 The upper limits of 95% prediction intervals (PI) of LVDd (A) and LVDs (B) as a function of body weight. The curves were generated by a GAM model of the 2 populations all nonsighthound dogs (red line, n = 6097) and immaculate nonsighthound dogs (green line, n = 1794). [file JVIM-34-2242-s001.pdf]
