# Supplementary material for: Left ventricular M‐mode prediction intervals in 7651 dogs: Population‐wide and selected breed‐specific values
Source: J Vet Intern Med. 2020 Oct 2;34(6):2242–52. doi: 10.1111/jvim.15914 (PMC7694859; doi:10.1111/jvim.15914)
Supplement: Supplementary file 2 — Supplementary Table 1 Number of dogs in each breed in all 7651 dogs (including sighthounds). Supplementary Table 2: Characteristics of dogs of breeds (n = 7003) in which echocardiographic measurements were compared to the 95% prediction intervals generated in the all nonsighthound group (n = 6097). Sighthound breeds are marked with an asterisk. N = Number of dogs, BW = Median body weight, Age = Mean age. Supplementary Table 3: Estimates of mean difference between the upper limits (of all measured variables LVDd, LVDs, IVSd, IVSs, LVWd and LVWs) of the groups all nonsighthound dogs (n = 6097) and immaculate nonsighthound dogs (n = 1794) based on a generalized additive model including a nonlinear effect of body weight. Supplementary Table 4: Estimated interobserver variability (in percent) based on the weight‐independent residual variance for the examined echocardiographic variables in the all nonsighthound group (n = 6097) [file JVIM-34-2242-s002.pdf]

**Supplementary Table 1:** Number of dogs in each breed in all 7651 dogs (including sighthounds).

| Breed | Number of dogs | Breed | Number of dogs |
|---|---|---|---|
| Afghan | 306 | Korea Jindo Dog | 1 |
| Airedale Terrier | 3 | Kuvasz | 3 |
| Alaskan Malamute | 1 | Labrador Retriever | 159 |
| American Bulldog | 27 | Leonberger | 4 |
| Antikdogge | 11 | Lhasa Apso | 1 |
| Australian Cattle Dog | 1 | Magyar Agar | 1 |
| Australian Kelpie | 9 | Malinois | 3 |
| Australian Shepherd | 8 | Malteser | 6 |
| Barsoi | 9 | Mastiff | 4 |
| Basenji | 2 | Mastin De Los Pirineos | 1 |
| Basset Hound | 1 | Mastino Napoletano | 1 |
| Beagle | 21 | Miniature Australian Shepherd | 5 |
| Bearded Collie | 3 | Miniature Dachshund | 1 |
| Belgian Shepherd Dog | 1 | Miniature Poodle | 2 |
| Berger Des Pyrenees | 11 | Miniature Schnauzer | 1 |
| Bernese Mountain Dog | 1 | Mittle German Spitz | 1 |
| Bichon Frise | 3 | Mudi | 1 |
| Bobtail | 2 | Newfoundland Dog | 161 |
| Border Collie | 8 | Owczarek Podhalanski | 2 |
| Boston Terrier | 2 | Papillon | 43 |
| Bouvier des Ardennes | 1 | Parson Russell Terrier | 11 |
| Boxer | 3111 | Phalene | 2 |
| Bracken | 11 | Pinscher | 4 |
| Broholmer | 4 | Pointer | 1 |
| Bullterrier | 71 | Polski Owczarek Nizinny | 121 |
| Cane Corso | 13 | Pug | 6 |
| Cavalier King Charles Spaniel | 94 | Puli | 3 |
| Chihuahua | 6 | Pumi | 5 |
| Chinese Crested Dog | 8 | Pyrenean Mountaindog | 7 |
| Collie | 2 | Rhodesian Ridgeback | 58 |
| Coton de Tuléar | 1 | Rottweiler | 8 |
| Dachshund | 18 | Saarloos Wolfhond | 1 |
| Dalmatian | 2 | Saint Bernard dog | 1 |
| Deerhound | 7 | Saluki | 302 |
| Doberman Pinscher | 427 | Samojede | 4 |
| Dogo Canario | 4 | Saupacker | 2 |
| Dogue De Bordeaux | 32 | Schapendoes | 1 |
| English Bulldog | 34 | Schnauzer | 12 |
| English Cocker Spaniel | 4 | Russian Black Terrier | 3 |
| English Springer Spaniel | 4 | Setter | 4 |
| Eurasian | 2 | Sheltie (Shetland Sheepdog) | 6 |
| Fila Brasileiro | 1 | Shih Tzu | 3 |
| Flat Coated Retriever | 7 | Siberian Husky | 2 |
| French Bulldog | 203 | Silken Windsprite | 25 |
| Galgo Espanol | 2 | Sloughi | 3 |
| Giant Schnauzer | 7 | Slovensky Kopov | 23 |
| German Shepherd Dog | 12 | Small Munsterlander Pointer | 1 |
| Golden Retriever | 89 | Stabyhoun | 1 |
| Great Dane | 900 | Staffordshire Bull Terrier | 3 |
| Greyhound | 2 | Standard Poodles | 4 |
| Havaneser | 1 | Suomenajokoira | 1 |
| Hovawart | 184 | Swiss Shepherd Dog | 9 |
| Irish Glen Of Imaal Terrier | 1 | Tibet Terrier | 1 |
| Irish Terrier | 4 | Welsh Terrier | 1 |
| Irish Wolfhound | 837 | West Highland White Terrier | 1 |
| Italian Greyhound | 13 | Whippet | 47 |
| Jack Russell Terrier | 3 | Wolfsspitz | 4 |
| Japanese Spitz | 1 | Yorkshire Terrier | 3 |
| Komondor | 2 | Total | 7651 |

**Supplementary Table 2:** Characteristics of dogs of breeds (n=7003) in which echocardiographic measurements were compared to the 95% prediction intervals generated in the all non-sighthound group (n=6097). Sighthound breeds are marked with an asterisk. N = Number of dogs, BW = Median body weight, Age = Mean age.

| Breed | N | BW | Age | Sex | |
|---|---|---|---|---|---|
| | | | | Female | Male |
| Afghan* | 306 | 27.0 | 4.6 | 57.5% | 42.5% |
| Barsoi* | 9 | 28.0 | 4.7 | 66.7% | 33.3% |
| Boxer | 3111 | 27.5 | 1.7 | 55.9% | 44.1% |
| Cavalier King Charles Spaniel | 94 | 8.4 | 3.1 | 60.6% | 39.4% |
| Deerhound* | 7 | 45.3 | 4.6 | 42.9% | 57.1% |
| Doberman | 427 | 36.0 | 4.2 | 55.3% | 44.7% |
| French Bulldog | 203 | 12.0 | 1.6 | 64.0% | 36.0% |
| Galgo Espanol* | 2 | 20.8 | 7.2 | 100.0 % | - |
| Golden Retriever | 89 | 29.7 | 2.7 | 51.7% | 48.3% |
| Great Dane | 900 | 63.5 | 3.6 | 61.2% | 38.8% |
| Greyhound* | 2 | 32.4 | 3.5 | 100.0% | - |
| Hovawart | 184 | 36.1 | 4.1 | 41.3% | 58.7% |
| Italian Greyhound* | 13 | 4.8 | 1.7 | 53.9% | 46.1% |
| Irish Wolfhound* | 837 | 60.0 | 3.2 | 65.6% | 34.4% |
| Labrador Retriever | 159 | 30.0 | 3.7 | 47.2% | 52.8% |
| Magyar Agar* | 1 | 24.0 | 7.6 | 100.0% | - |
| Newfoundland Dog | 161 | 53.7 | 3.1 | 60.9% | 39.1% |
| Polski Owczarek Nizinny | 121 | 18.1 | 2.7 | 57.0% | 43.0% |
| Saluki* | 302 | 22.4 | 4.4 | 54.6% | 45.4% |
| Silken Windsprite* | 25 | 11.4 | 3.8 | 52.0% | 48.0% |
| Sloughi* | 3 | 28.0 | 3.3 | 33.3% | 66.7% |
| Whippet* | 47 | 13.0 | 2.9 | 51.2% | 48.8% |

**Supplementary Table 3:** Estimates of mean difference between the upper limits (of all measured variables LVDd, LVDs, IVSd, IVSs, LVWd and LVWs) of the groups *all non-sighthound dogs (n=6097)* and *immaculate non-sighthound dogs (n=1794)* based on a generalized additive model including a non-linear effect of body weight.

| | Estimate | Standard Error | P-value |
|---|---|---|---|
| Intercept | 2.460 | 0.087 | <0.001 |
| All non-sighthound dogs | 0.009 | 0.123 | 0.942 |

**Supplementary Table 4**: Estimated interobserver variability (in percent) based on the weight-independent residual variance for the examined echocardiographic variables in *the all non-sighthound group (n=6097).*

| Parameter | Interobserver-Variability in percent |
|---|---|
| LVDd | 10.2 |
| LVDs | 8.1 |
| IVSd | 18.8 |
| IVSs | 24.0 |
| LVWd | 30.6 |
| LVWs | 20.6 |
